# New Approaches to Assess Mechanisms of Action of Selective Vitamin D Analogues

**DOI:** 10.3390/ijms222212352

**Published:** 2021-11-16

**Authors:** John Wesley Pike, Mark B. Meyer

**Affiliations:** Department of Biochemistry, University of Wisconsin-Madison, 433 Babcock Drive, Madison, WI 53706, USA; markmeyer@wisc.edu

**Keywords:** vitamin D biology and action, transcription, ChIP-chip analysis, distal enhancers, histone H3 acetylation, RNA polymerase II, analogue actions at genes, vitamin D hormone (1,25(OH)_2_D_3_)

## Abstract

Recent studies of transcription have revealed an advanced set of overarching principles that govern vitamin D action on a genome-wide scale. These tenets of vitamin D transcription have emerged as a result of the application of now well-established techniques of chromatin immunoprecipitation coupled to next-generation DNA sequencing that have now been linked directly to CRISPR-Cas9 genomic editing in culture cells and in mouse tissues in vivo. Accordingly, these techniques have established that the vitamin D hormone modulates sets of cell-type specific genes via an initial action that involves rapid binding of the VDR–ligand complex to multiple enhancer elements at open chromatin sites that drive the expression of individual genes. Importantly, a sequential set of downstream events follows this initial binding that results in rapid histone acetylation at these sites, the recruitment of additional histone modifiers across the gene locus, and in many cases, the appearance of H3K36me3 and RNA polymerase II across gene bodies. The measured recruitment of these factors and/or activities and their presence at specific regions in the gene locus correlate with the emerging presence of cognate transcripts, thereby highlighting sequential molecular events that occur during activation of most genes both in vitro and in vivo. These features provide a novel approach to the study of vitamin D analogs and their actions in vivo and suggest that they can be used for synthetic compound evaluation and to select for novel tissue- and gene-specific features. This may be particularly useful for ligand activation of nuclear receptors given the targeting of these factors directly to genetic sites in the nucleus.

## 1. Background and Review Rationale

The fundamental actions of the steroid hormone 1,25-dihydroxyvitamin D_3_ (1,25(OH)_2_D_3_) are to contribute to the maintenance of calcium (Ca) and phosphorus (P) homeostasis in vertebrate organisms [1]. This activity is achieved through direct actions of the hormone in the intestine, kidney, and bone, and through feedback inhibition of PTH production at the parathyroid glands and induction of FGF23 production in mature osteoblasts and osteocytes. In the intestine, the transepithelial uptake of Ca is known to be upregulated by 1,25(OH)_2_D_3_ via induction of the apical Ca ion channel gene *TRPV6*, genes encoding the soluble cytosolic Ca binding calbindins, and genes that include the basolateral ATPase driven Ca pump *PMCA2b*. P uptake, on the other hand, is regulated in the intestine by induction of the P transporter gene *SLC34A2* [2]. In the kidney, Ca levels are controlled directly via the induction of TRPV5, the calbindins, and the sodium/Ca exchanger *NCX1*, while P is regulated by *SLC34A1* and *SLC34A3* and indirectly via 1,25(OH)_2_D_3_’s regulatory control of hormonal PTH and FGF23 [3,4,5,6]. FGF23 is dominant with regard to P regulation, although PTH has similar yet more modest actions [7,8]. Activity in the skeleton is driven via 1,25(OH)_2_D_3_ and PTH, primarily via RANKL expression, a TNF-like factor that is produced in stroma, osteoblastic cells, and osteocytes and is essential to the formation, activation, and survival of bone-resorbing osteoclasts [9,10,11]. Recent studies suggest that the osteocyte plays a fundamental role in lacunar bone formation and resorption within cortical bone as well [12]. The vitamin D hormone also contributes to the regulation of many additional genes that are involved not only in osteocyte function, as above, but also in osteoblast differentiation, bone formation, and biomineralization [13,14,15]. This hormone also contributes to the transcriptional regulation of *FGF23*, a primarily osteocyte-produced hormone [16,17,18]. While the function of the vitamin D hormone in the kidney (we use 1,25(OH)_2_D_3_ and the vitamin D hormone interchangeably) involves genetic control of Ca and P reabsorption, as described above, its actions also extend to many additional genes [19,20]. Indeed, a primary function of 1,25(OH)_2_D_3_ is to regulate the expression of renal *Cyp27b1* and *Cyp24a1*, two genes that, in the kidney, are responsible for the synthesis as well as turnover of 1,25(OH)_2_D_3_ [21]. This regulation occurs by not only 1,25(OH)_2_D_3_ but also by FGF23, both of which suppress *Cyp27b1* expression and induce *Cyp24a1*. These two genes are also regulated by PTH, which induces *Cyp27b1* while suppressing *Cyp24a1* [22]. These three hormones represent the primary determinants of circulating 1,25(OH)_2_D_3_ in the blood. Individual disruption or loss of expression of any of these hormones, either via genetic manipulation, altered hormonal metabolism, or through disease, results in a significant impact on extracellular Ca and/or P levels in vivo, supporting their essential role in the regulation of mineral homeostasis.

In the past decade or more, however, the actions of vitamin D have also been increasingly linked to a diverse set of important biological activities in non-renal tissues that appear to be separate from those that are involved in mineral regulation [23]. In many of these targets and specific cell types within, the actions of vitamin D in vitro appear to contribute to differentiation and/or maturation and to the regulation of genes involved in those processes. Cell types include those of the immune system, but also those in skin, the cardiovascular system, including smooth muscle cells and cardiac cells, striated muscle cells, and diverse reproductive and placental tissue targets, to name only a few [24,25,26]. Consistent with the validity of these assertions, these cell types retain the molecular machinery essential for mediating 1,25(OH)_2_D_3_ regulation through genes that are essential for unique biological responses (see below). It has also been suggested that many of these cell types may also synthesize and catabolically degrade 1,25(OH)_2_D_3_ locally via *Cyp27b1* and *Cyp24a1*, respectively [27]. Despite the very low levels of expression of these two genes in non-renal tissues, the observations suggest a secondary pathway as an entrée into an additional sphere of vitamin D biology that could potentially be independent of that which maintains circulating endocrine 1,25(OH)_2_D_3_. In support of this concept, the regulation of *Cyp27b1* in non-renal cell types differs substantially from that in kidney. Accordingly, this gene is regulated not by PTH, FGF23, or 1,25(OH)_2_D_3_, as in kidney, but rather by a variety of inflammatory cytokine modulators [28,29,30]. *Cyp24a1*, on the other hand, is not regulated in these tissues by either PTH or FGF23 as in the kidney but remains sensitive to 1,25(OH)_2_D_3_ [30]. This suggests that the primary functional role for *Cyp24a1* in these cells may be to prevent 1,25(OH)_2_D_3_ toxicity. These differences in regulation support the idea that local production of 1,25(OH)_2_D_3_ may function in these cell types in a fashion distinct from those involved in mineral metabolism. While much needs to be learned about the impact of the local production of 1,25(OH)_2_D_3_, the diverse actions of the vitamin D hormone in this broad array of tissue/cellular targets supports the concept that vitamin D may play a contributory role in biological processes that extend beyond mineral metabolism. They also imply that vitamin D deficiency could have a unique impact on these numerous biological processes. Thus, restoration of the parent vitamin or the addition of 1,25(OH)_2_D_3_ and/or a selective analogue of 1,25(OH)_2_D_3_ could be particularly useful therapeutically in diseases such as those of altered immune function or in cancer, where vitamin D may exert beneficial actions. Indeed, by promoting differentiation, 1,25(OH)_2_D_3_ not only reduces tumor cell growth but may also function to reduce inflammation through the down-regulation of inflammatory mediators [31]. While attractive conceptually, validation of these hypotheses in human disease requires further confirmation.

Considering the possibility that analogues of vitamin D and/or 1,25(OH)_2_D_3_ might be designed to selectively target tissues involved in these secondary biology activities of vitamin D, chemists have synthesized and examined hundreds of biologically active vitamin D-like compounds for tissue-selective activity over several decades or more. The identification of compounds potentially useful therapeutically has, however, met with generally limited success. We posit that this may be due to the lack of rigorous structure/function assays in vivo that are necessary for guiding the efforts of synthetic chemists. In this article, we suggest that advances in our understanding of vitamin D action at the transcriptional level in vivo may provide not only useful structure/function relationships, but also detailed genomic analyses that could well identify the underlying mechanisms through which a novel analog might operate to manifest tissue, cell, and gene selectivity. Thus, this article represents a combination of transcriptional review, opinion piece, and proposal for incorporating new methods into the analysis of both existing as well as de novo synthesized new vitamin D chemical entities.

## 2. Transcriptional Mechanism of Action of 1,25(OH)_2_D_3_

Early studies using radioactive ligands served to both identify the existence of the VDR and to facilitate preliminary characterization and purification of the protein itself, an approach that also led similarly to the characterization of nuclear receptors (NR) for other soluble endocrine hormones [32]. It was the cloning of the VDR, however, that placed it unequivocally within the emerging family of concurrently cloned NRs [33,34,35]. It also precipitated the field’s move towards true molecular studies that eventually identified the protein’s structural organization, key functional domains, and ultimately, its 3D crystallographic structure [36,37,38]. As these discoveries evolved, however, additional research led to the realization that, in contrast to the true steroid receptors, the VDR formed a 1,25(OH)_2_D_3_-dependent heterodimer with a nuclear accessory factor (NAF) that was required for DNA binding; subsequent experiments proved that this factor was RXR, a member of the NR family composed of three isoforms [39,40,41,42,43]. These studies also identified the first DNA response element (VDRE) that served as a binding site for the VDR; this site was located near the promoter within the human osteocalcin gene [41,42,43]. While many VDREs have been identified since, initially through traditional transfection methods and currently through genome-wide VDR/RXR DNA binding studies using ChIP-seq analysis, this single promoter-proximal element in the osteocalcin gene has been repeatedly confirmed [15]. In total, this collection of early molecular biological studies revealed that NRs including the VDR function largely facilitate the formation of complex multi-protein structures at VDRE-containing sites on vitamin D target genes. These protein complexes contain distinct members of several classes of transcriptional co-regulators capable of modifying chromatin structure and/or directing functions that are necessary for either the induction or the suppression of transcriptional output [44,45]. Many of these latter factors do not bind to DNA directly, however, but establish contact via protein–protein interactions. Thus, similarly to other NRs and most DNA binding transcription factors, the VDR serves as an initial site-specific DNA adaptor, although there are hints that it may also sub-serve a non-DNA binding role by interacting with proteins previously bound to DNA as well.

## 3. Analogues of Vitamin D

The biological actions of vitamin D to control mineral metabolism prompted the early synthesis of 1,25(OH)_2_D_3_ and initial investigations into the utility of this hormone as a therapeutic for multiple diseases. Many of these maladies altered vitamin D metabolism and thus provoked crucial deficiencies in extracellular Ca and/or P that resulted in loss of bone mineral density and skeletal abnormalities. Classic genetic diseases included vitamin D dependent rickets type 1, later identified as arising from a mutation in the renal *Cyp27b1* gene that causes crippling 1,25(OH)_2_D_3_ deficiency due to an inability to hydroxylate 25(OH)D_3_ at the 1α position, and vitamin D-resistant rickets due to mutations in the VDR [46]. Much of this discovery work was prompted early on by the development of clinically relevant assays for blood 1,25(OH)_2_D_3_, pioneered initially by Haussler and his colleagues [47]. The therapeutic utility of 1,25(OH)_2_D_3_ was limited in many instances, however, by its potency and narrow therapeutic window, which frequently prompted hypercalcemia through predictable actions in intestine, kidney, and bone. These clinical difficulties prompted strong efforts by chemists to synthesize analogues of vitamin D and/or 1,25(OH)_2_D_3_ that were of greater practical utility, and perhaps to identify analogues with reduced “side-effects” relative to hypercalcemia. Reviews of these efforts and the many analogues that were synthesized have been documented extensively over several decades [48]. While they will not be discussed here, synthesis is ongoing in many laboratories. As biological effects of 1,25(OH)_2_D_3_ that were separate from those influencing mineral metabolism (growth, differentiation, immune effects) became evident, chemists attempted to home in on the creation of analogues with preferential biological activities that lacked calcemic activity. These selective disease targets included those related to chronic kidney disease and its eventual consequences on the skeleton and the cardiovascular system, as well as on hyperproliferative disorders that included cancer [49,50,51,52,53]. In retrospect, however, while numerous analogues appeared to retain a subset of these properties, the analyses that supported them were frequently misinterpreted. Consequently, vitamin D analogues have not proven to be particularly effective in targeted, non-calcemic biology, and no real understanding has emerged as to the mechanisms that might underpin the purported actions of these “non-calcemic” compounds. The absence of key assays for these analogues, based upon rigorous structure/function/activity relationships (SAR) either in vitro or in vivo, has exacerbated the efforts expended over the years to synthesize analogues with these properties. Indeed, basic and clinical scientists now have thousands of vitamin D analogues available for study. However, a rather limited understanding of the structural basis for any of the functional properties ascribed to these analogues has emerged, and no real insight has been gained as to their mechanisms. Consequently, only a few of the many analogues synthesized by chemists have become useful therapeutics, and those that have emerged have frequently exploited unique approaches to their administration. Interestingly, even the basic tenant that vitamin D supplementation in clinical trials should result in improved metabolite levels in patients deficient in vitamin D, and where additional 1,25(OH)_2_D_3_ was predicted to ameliorate disease onset (cancer, cardiovascular disease, prediabetes, and tuberculosis to name a few) has not been particularly effective in clinical trials [54,55,56,57,58]. A most recent example is the utility of vitamin D supplementation in the prevention of SARS-CoV2 disease, despite the fact that vitamin D has been shown in basic studies to impact the very molecular targets that facilitate viral cellular entry [59]. One might imagine that, until vitamin D analogues can be developed based upon rational SAR and their mechanisms firmly established in vivo, an entry point for their therapeutic use is not likely to emerge.

## 4. Pharmacology of Vitamin D Analogues

How might analogues operate mechanistically? The possibilities are enormous and include such features as the individual pharmacologic, pharmacokinetic, and/or pharmacodynamic properties of the compounds themselves. Speculatively, these also include parameters impacting the distribution of the compounds based upon their interaction affinity with vitamin D binding protein (DBP) and other serum-interacting factors, requirements for activation by residual renal and non-renal enzymes, and metabolic outcomes that could lead to retention of residual vitamin D-like activity. They also include the potential for selective tissue distribution and localization, relative rates of tissue uptake, as well as unique metabolic degradation rates determined either through catabolic vitamin D enzymes or by novel degradative enzymes not known routinely to degrade vitamin D compounds. With regard to the VDR, the relative biological activity of an analogue is almost certain to be affected by its affinity for the receptor, the longevity of the ligand receptor complex at its sites of action on DNA, and/or the compound’s novel impact on VDR structure. The latter could uniquely alter receptor function at numerous downstream mechanistic levels prior to or while bound to DNA at target gene loci, thereby altering the transcriptional outcome in a potentially selective manner. While the impact of ligand-dependent pharmacodynamic issues has only briefly been considered, the impact of novel ligands on VDR structure has been an attractive hypothesis for years, and has garnered much attention, largely due to the possibility that altered VDR structure mirrors that seen for several other steroid receptors, particularly the estrogen receptor [60,61,62,63,64,65,66,67,68]. Indeed, the structure of this receptor has been the focus of much attention, since it displays differential activity depending upon the tissue involved, and this appears to correlate with altered 3D crystal structures of ER. It is noteworthy, however, that this type of differential activity of the VDR on vitamin D biology has not been identified. Accordingly, the evidence supporting the impact of novel vitamin D analogues on the VDR protein is indirect, modest at best, and based largely upon techniques and data generated exclusively in vitro almost three decades ago [69,70]. While additional efforts have been made to identify novel ligand-induced anomalies at the VDR protein utilizing 3D crystallography, the results of these studies have been limited to the utilization of the ligand-binding portion of the VDR, which together with crystal structure elucidation may or may not reflect true structural changes in the holoprotein [71,72]. In addition, analogue-based studies have not been conducted in combination with RXR in the presence of appropriate DNA or in the presence of additional cofactors required for transcriptional regulation. Perhaps most importantly, with the exception of antagonist-induced loss of function, none of these structural differences have been linked directly to any selective differences in vitamin D biology at genes in vivo. Indeed, early attempts at linking VDR activity to transcription was limited to molecular biologic studies that are now viewed as generally inconsistent with concepts that have emerged over the past decade and that occur at the level of endogenous genes in a natural genomic environment. Finally, it is also not clear whether ligand affinity measurements determined in in vitro biochemical assays to assess analogue interaction relative to 1,25(OH)_2_D_3_ are impacted upon the formation of active VDR complexes on DNA within the nucleus of cells. We will discuss new ways below, wherein altered or disrupted actions of the VDR at individual genes and in different tissues might be determined not only in vitro but also in vivo.

## 5. New Insights into Transcriptional Activation of Vitamin D

The development of chromatin-immunoprecipitation and other genomic procedures coupled to next-generation DNA sequencing technics (ChIP-seq analysis) and their applications have fully changed the way in which gene regulation is evaluated. This technique has widespread applications, including the capacity to identify and characterize ligand-induced transcription factor binding activity, coregulator recruitment to specific genomic sites, and to assess downstream actions such as RNA polymerase II recruitment in a generally unbiased, genome-wide fashion [73]. These studies have also revealed a multitude of new details inherent to transcriptional processes at a genome-wide scale; some of these principles confirm what has previously been determined, while others identify modifications of those already known to exist. These methods have also revealed entirely new principles of gene regulation at both genome-wide as well as single-gene levels that need to be considered. Perhaps just as important, they have also revealed how inherent technical bias now disqualifies many of the conclusions drawn from over three decades of analyses using in vitro molecular biological methods involving transfection. With regard to the molecular actions of vitamin D, genome-wide analyses using chromatin immunoprecipitation, as summarized in Table 1, has revealed many relevant findings. As can be seen, these analyses in both human and mouse cultured cells as well as tissues in vivo indicate between 2000 and 8000 VDR binding sites; the number and location of these sites are affected by the quality of the analyses, but within a single laboratory, can be species and cell type specific [45,74,75,76,77]. Most of, but not all, these sites are highly dependent upon 1,25(OH)_2_D_3_ pretreatment, indicating that VDR represents a ligand-dependent DNA binding factor. RXR DNA binding is also modestly induced by 1,25(OH)_2_D_3_ at these VDR occupied sites as well, although RXR can be residually present even in the absence of ligand. This has suggested that prebound RXR may serve an initial role, perhaps to facilitate (affinity) and/or mark specific DNA sites for subsequent VDR binding that is dependent upon DNA half-site configuration. As RXR interacts with numerous additional NRs, it is also possible that RXR may exist at some of these sites with an alternative partner [78]. Nevertheless, the bioinformatically determined close proximity of VDR and RXR at most genomic sites reinforces the idea that the active transcription factor complex is indeed the VDR/RXR heterodimer [14,15]. De novo sequence analysis across these VDR sites confirms the presence of at least one and often multiple motifs, each composed of a typical VDRE of two hexameric DNA half-sites separated by three base pairs. This DNA structure represents a typical VDRE currently at hundreds of genes and in all species examined, although one of the half-sites in a VDRE is frequently highly degenerate, making it difficult in some cases to identify this motif. Additional DNA motifs with dissimilar nucleotide arrangements are also evident at some sites and may represent additional VDR/RXR interacting sequences yet to be explored. In addition, VDR binding at sites known to function in repression often contain novel DNA motifs, suggesting that the VDR may be bound to these sites indirectly via association with other DNA or non-DNA binding proteins that serve additional regulatory functions. These observations at genome-wide levels both confirm and extend our understanding of several key features of vitamin D action via the VDR at target genes. Perhaps the most important discovery arising from unbiased genome-wide analyses of VDR/RXR binding sites and, indeed, most other transcription factor-binding sites is the observation that these regulatory sites are not located exclusively near target gene promoters as previously suspected [22,74,79]. Rather, they can be found at sites that are commonly distributed within intergenic regions both upstream and downstream of the genes they regulate, often with unregulated genes interspersed, as well as commonly within the introns of the target gene itself or in unregulated genes located adjacent to the regulated gene or both. These can also be located 10s if not 100s of kilobases distal to a gene’s promoter as well, the locations of which may have an impact on their function [80,81,82,83]. Perhaps even more interestingly, this regulatory capacity is frequently composed of multiple interacting components, often 2 to 10 or more regulatory regions, each representing an individual and perhaps unique enhancer function. Finally, these individual enhancers are also modular in nature, serving to bind multiple factors that can span a kilobase or less to more complex segments that can span many kilobases; super-enhancers and other large regulatory regions to which the VDR binds, appear to represent consolidated collections of multiple enhances that can span over 20 kilobases [84].

Since multiple enhancers may control a single gene, and the localization of transcription factors at sites on the genome does not identify the gene or genes they regulate, it is certain that enhancer location near a gene cannot be used to determine a gene target. Accordingly, the genome-wide number of VDR binding sites in a cell, for example, is not useful in determining the number or identity of genes that are regulated by the hormone. Indeed, the repeated suggestion that the VDR regulates a high percent of genes in the genome is highly misleading, since the VDR binds in a cell-specific manner to only small, although overlapping, subsets of genes in any given cell type. Furthermore, temporal delays in regulation suggest that many if not most purported target genes for vitamin D likely result from indirect secondary and tertiary activity. These evolve from vitamin D’s traditional capacity to induce autocrine and paracrine growth factors and/or endocrine hormones as well as other components that influence in turn the activity of multiple cellular signaling pathways. It would also appear that the activation of some rapidly induced genes are temporally consistent with a direct action, yet represent activities of a secondary nature as well, as appears to be the case for FGF23 [13]. Details of this type of activity remain unclear. Importantly, current knowledge also indicates that the functional outcomes of transcription factor binding and coregulator recruitment can be evaluated via epigenetic modifications that are now measurable at genes via current technics [85,86]. Some of these epigenetic modifications are linked directly to the facilitation of transcriptional output [85]. Mechanistically, it is also worth noting that, although linear arrangements of binding sites at a gene locus are often schematically displayed, the actual arrangement in vivo is almost certainly a 3D complex of all or subsets of these regulatory units via looping. Interestingly, these enhancer regions are also characterized by an open chromatin structure that is assembled ultimately near target genes and serves in an integrative fashion to control the transcriptional output of the gene itself. These enhancers may also retain a unique historical, temporal, and spatial function, conferring regulatory capacity on a subset of cell types in a timely manner. This complexity also indicates why earlier studies using cell lines transfected with biased gene promoter plasmid constructs with or without transcription factor or coregulator cotransfection has resulted in numerous conclusions about the regulation of individual genes that fails to confirm at endogenous genes with newly devised, unbiased ChIP-seq methods [29,30,74]. Finally, as indicated earlier, it is noteworthy that binding sites for transcription factors such as the VDR are not fixed, but are dynamic, even within a cell type. Thus, they can be altered via epigenetic modification to accommodate the cell’s differentiation and/or maturation status, physiological environment, or perhaps more importantly, the host’s state of health or disease [87].

## 6. Current Advances in Transcriptional Regulation

The discovery of novel pathways, as well as insights into the mechanisms of existing and now well-described regulators of transcription, provide reliable avenues through which mechanistic advances can be made and new compounds with drug potential can be synthesized and assessed. Certainly, the overarching principles described in Table 1 that now describe transcriptional regulation by vitamin D are also applicable to regulation by additional small molecules. Accordingly, they have led to new methods to characterize the details of gene regulation both in vitro and in vivo, the latter at molecular levels almost equivalent to those heretofore achievable only in cultured cells. Indeed, many of the details from these studies have been enhanced in vivo through recent analyses of selected cell populations from complex tissues and from the development of genome-wide techniques with increased sensitivity [88]. Organoids created from embryonic stem cells have also provided new in vitro insight as well, although cellular mass for analysis is frequently limited. Details now extend to the coupling of changing epigenetic architecture in response to genetic and epigenetic action at regulatory loci to the recruitment of additional transcription factors that play direct roles in promoting or inhibiting transcriptional output. These include detection of epigenetic readers that affect the distribution and release of RNA polymerase II across the transcription unit to those that influence the downstream mechanisms essential for transcription of the gene per se, resulting in the production of RNA transcripts [85,89]. The capacity to detect the presence and activity of many of these factors at genes and to assess the potential consequences of these factors at both genetic and epigenetic levels is important. It suggests that transcriptional mechanisms for 1,25(OH)_2_D_3_ that result from the binding of the VDR to its site(s) of action can be assessed, and that detailed consequences arising from the activity of prospective vitamin D analogs may also be determined in an endogenous gene context. Thus, temporal relationships, tissue and cellular specificity, and gene selectivity and coregulator recruitment specificity (or lack thereof) can all be determined directly at this endogenous level. Of additional advantage is the fact that these studies can frequently benefit through an assessment of the overarching functional roles of specific enhancers deleted at the genes of interest using CRISPR/Cas9 methods in cells and in animals in vivo [90,91]. Thus, new actionable opportunities have now emerged to determine the mechanisms through which existing as well as newly synthesized vitamin D analogues operate as compared to 1,25(OH)_2_D_3_. These assays might now provide a rational SAR approach for driving the synthesis of new vitamin D analogues with directed tissue activities as well.

## 7. Recent Work at the Frontier for Enhancing Vitamin D Analogue SAR Potential

We have taken a pragmatic approach to the study of gene expression and its regulation by 1,25(OH)_2_D_3_. This effort focused initially upon a variety of cell types in vitro, where we identified many of the principles enumerated in Table 1. Subsequently, however, we have focused on unique cell types in culture using the above approach and then transitioned to parallel studies in vivo using rodents. Insights gained from the initial studies in cells facilitated this transition, providing baseline insight that has enabled in vivo studies to commence at a mechanistic level equivalent to those conducted in vitro. The advantages of in vivo studies are numerous, but first and foremost, they are free of the bias that has limited those conducted in cultured cells. Secondly, they enable concurrent analyses of molecular mechanisms at multiple genes that are potentially regulated and expressed uniquely in different target tissues. This advantage facilitated the study of the differential regulation of specific genes, the influence of cellular background, and the identification of mechanisms that underlie these differences as well. Most recently, this approach has been strengthened through the ability to exploit emerging methodologies. These techniques can also be used on a genome-wide basis to isolate specific cell types and single cells in order to delineate the sources and molecular features of the cells that are involved in the expression of genes of interest [92,93,94]. Finally, loss of function/regulation studies utilizing CRISPR/Cas9 mediated gene-editing methods can be employed in vivo to determine the regulatory impact of removing key enhancer regions as well as specific DNA sequences within the loci of genes of interest in order to map regulatory regions in response to specific systemic regulators [29,30,90].

**Figure 1:**
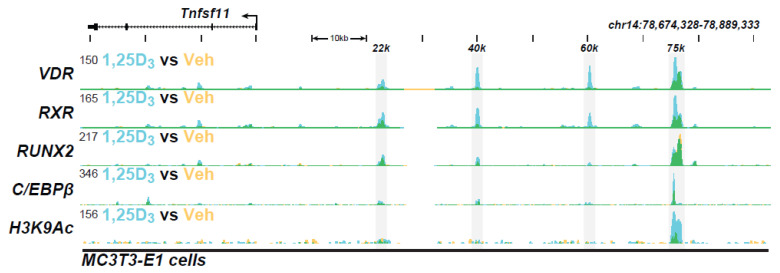
The ChIP-seq tracts for *Tnfsf11* gene were derived from a previous genome-wide analysis of the IDG-SW3 osteocyte cell line in Ref. [13].

**Bone genes:** We first explored the genome-wide DNA binding site profile (termed a cistrome) for VDR and RXR in osteoblastic and osteocytic cell lines. These initial studies as well as others highlighted the interaction of the VDR at multiple sites near many genes involved in bone cell maturation, bone formation, biomineralization, and osteoclast-mediated bone resorption. As an example, we initially explored the effects of 1,25(OH)_2_D_3_ on the *Tnfsf11* gene for RANKL in both cell types [13,95,96]. As can be seen in the ChIP-seq tracks in Figure 1, 1,25(OH)_2_D_3_ induced the localization of both VDR and RXR to four regulatory regions that extended upstream of the gene to 76 kb. Neither VDR nor RXR bound to regions near the *Tnfsf11* gene promoter. Additional factors bound to a subset of these enhancers including a master bone regulator RUNX2 and a potential pioneering factor CEBPβ [97]. These factors, as well as others, frequently colocalized to VDR/RXR binding sites at numerous additional genes as well. Importantly, deletion of several of these enhancers, including the distal 75 kb *Tnfsf11* segment in mice in vivo, resulted in a blunting of both 1,25(OH)_2_D_3_ and PTH induction [98]. Deletion of a region immediately upstream of the *Tnfsf11* promoter had no effect, confirming that this gene was not induced through promoter-proximal sequences [74]. Thus, the regulation of *Tnfsf11* by these two hormones is achieved through multiple distal enhancers. Many additional genes have been explored in bone cells in this manner including the *VDR* itself, *Mmp13*, *Lrp5*, and *Enpp1* to name a few. These and other studies therefore reveal roles for not only VDR and RXR, but other factors that are essential to 1,25(OH)_2_D_3_ action as well.

In osteocytes, the activity of 1,25(OH)_2_D_3_ was also linked to those regulated by PTH through the frequent presence CREB at VDR/RXR binding sites [96]. These studies also revealed a role for 1,25(OH)_2_D_3_ in enhancing the expression of two co-receptors essential to the Hedgehog pathway activity. This latter observation illuminated an example of the crosstalk that is provoked by 1,25(OH)_2_D_3_ and that is likely to initiate transcriptional effects secondary to those that are direct. Sites of VDR action were also characterized by the presence of active VDREs and classic epigenetic histone signatures that were indicative of dynamic regulatory chromatin architecture. At the *Tnfsf11* gene, as seen in Figure 1, the activity of the VDR/RXR heterodimer-induced histone H3K9 acetylation at the 75 kb region, indicating that VDR binding was active at this site for *Tnfsf11* expression. In many genes, all the above events, including co-localization of coregulators, the presence of RNA polymerase II, and enrichment of H3K36me3, a histone mark present at the gene body itself and reflective of the induction of transcripts, were also seen. These features are linked directly to the transcriptional output of genes themselves. Thus, they provide direct evidence for rapid 1,25(OH)_2_D_3_ modulated transcript output that can be detected at the genomic level and correlated via RNA analysis. Finally, the impact of many of the regulatory features identified for some of the bone genes listed above were explored in the mouse in vivo through loss of expression studies, wherein regulation of the expression of the gene of interest was eliminated entirely upon deletion of the specific enhancer region. These results show that the identified enhancers and the activities they engender are linked directly to specific genetic output in vivo. This demonstration is essential, since, as indicated earlier, enhancers may be located many kilobases (kb) distal to the genes they regulate. It also provides additional opportunities to understand how non-vitamin D regulators participate with 1,25(OH)_2_D_3_ in the expression of specific bone genes. We would anticipate that vitamin D analogues would show alteration in one or more of these progressive events during activation in vivo, perhaps in a tissue-specific manner.

Interestingly, the successful study of VDR/RXR binding across several bone cell genomes failed to identify the presence of either VDR or RXR across the extended locus that surrounds the *Fgf23* gene, despite the fact that 1,25(OH)_2_D_3_ induces *Fgf23* transcripts in these cells [13]. This observation provides significant evidence that the induction of *Fgf23* by 1,25(OH)_2_D_3_ may be indirect, perhaps through a primary DNA binding factor(s). One approach is to conduct enhancer deletion experiments in the mouse in vivo. Thus, an enhancer region that plays an extended role in 1,25(OH)_2_D_3_ induction of Fgf23, as established in vivo, could be dissected via loss of regulation studies to identify a more restricted genomic segment capable of mediating 1,25(OH)_2_D_3_ induction [99]. This approach extends the utility of in vivo studies to the level of fine genomic dissection of existing enhancer regions based upon DNA sequence. This functional approach can also reveal motifs that might lead to the identification of mediators of secondary actions of 1,25(OH)_2_D_3_.

**Figure 2:**
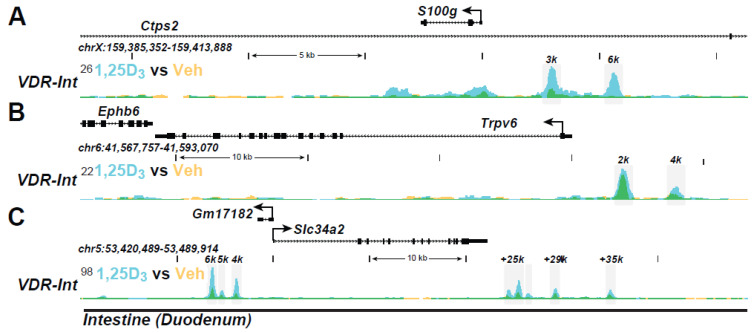
The ChIP-seq tracts for the intestinal genes (**A**) *Trpv5*, (**B**) *Trpv6*, and (**C**) *S100g* were derived from a previous genome-wide analysis of mouse intestinal epithelial cells in vivo as in Ref. [95].

**Intestinal epithelial genes:** Aside from functional studies, it has been the use of ChIP-seq analyses that has enabled a complete transition to the evaluation of gene regulation in vivo. Indeed, in addition to the advantages listed earlier, this approach provides opportunities for study at both tissue/cell- and genome-wide levels that are unparalleled. It may also facilitate a detailed study of regulation at genes of therapeutic interest using mouse disease models. 1,25(OH)_2_D_3_ action in vivo has been explored at different mechanistic levels in intestinal epithelia and in kidney cortex (enriched in proximal tubules). In intestinal epithelial cells isolated from mice pre-injected with 1,25(OH)_2_D_3_, the VDR is bound to multiple genes across the mouse genome; its binding is enhanced by prior treatment with 1,25(OH)_2_D_3_ [100,101,102]. More recent studies have revealed differences in VDR binding in enterocytes vs. crypt cells, suggesting differential roles for vitamin D in these two cells [101,102]. Enhancer regions at key genes in epithelial cells that are involved in calcium absorption are highlighted in the ChIP-seq tracks documented in Figure 2A,B, for *S100g* (calbindins D9k) and *Trpv6*; others are linked to intracellular Ca regulation and to the transport of elements such as manganese. The enhancers for *Trpv6* were validated previously in a human intestinal cell line. VDR/RXR also induced the intestinal P transporter *Slc34A2* (Figure 2C) as well. Contemporary knowledge of the sites of action of the VDR will now facilitate an up-to-date discovery that could reveal the identities of additional factors that regulate the transcriptional output of these genes and the role of epigenetic histone modifications in this process. This would enable mechanistic study of vitamin D analogues in vivo, purported to be deficient in their capacity to promote intestinal Ca and P uptake. In these cases, we might anticipate that certain “non-calcemic” analogues might exhibit reduced cofactor recruitment and perhaps histone acetylation.

**Figure 3:**
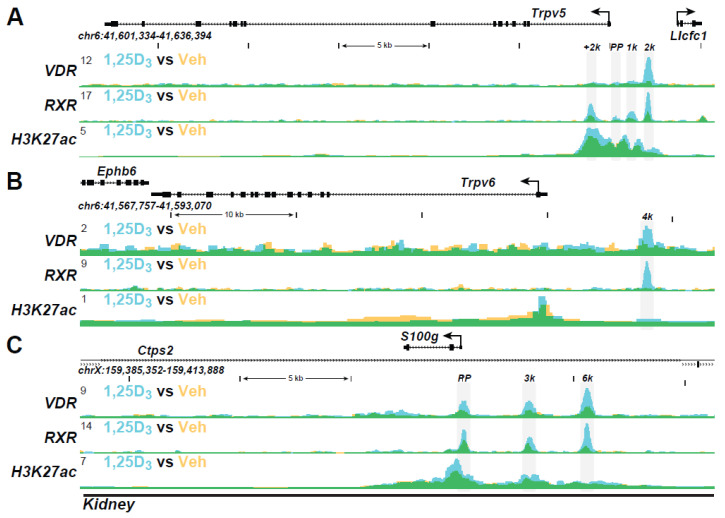
The ChIP-seq tracts for the kidney genes (**A**) *Trpv5*, (**B**) *Trpv6*, and (**C**) *S100g* were derived from a previous genome-wide analyses of mouse kidney cortex in vivo as in Ref. [29].

**Kidney Genes:** Perhaps the most extensive analysis of the mechanistic actions underpinning 1,25(OH)_2_D_3_ action in vivo have been conducted recently in the kidney. Multiple genes represent targets of vitamin D action in this organ. Indeed, multiple VDR and RXR binding sites are evident at *Trpv5*, *Trpv6*, and *S100g*, as seen in the ChIP-seq tracks in Figure 3A–C, supporting 1,25(OH)_2_D_3_ actions that are involved in Ca regulation in the kidney. Epigenetic chromatin activity is also seen at these sites for H3K27ac modification. VDR binding sites are also present upstream of the *Slc34a1* and *Slc34a3* genes that encode transporters involved in P reabsorption. Fgf23 displays actions in the kidney to regulate P transporter location, thus controlling P diuresis. These actions involve specific isoforms of the FGFRs, whose ability to bind Fgf23 is influenced by *Klotho*. The ability to dissect the many players involved in the regulation of genes in the kidney, bone, and intestine that are involved in the maintenance of Ca and P homeostasis is important. It suggests that they may prove useful in identifying unusual features of vitamin D analogs that have direct non-calcemic and/or P diuretic properties that may occur in a tissue-selective manner.

**Figure 4:**
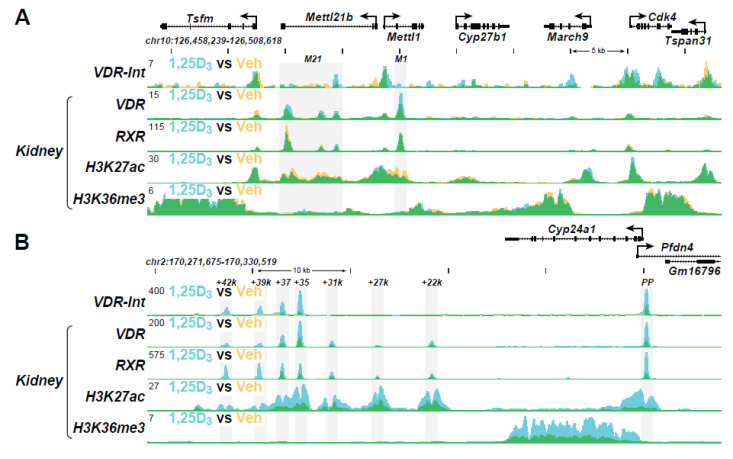
The ChIP-seq tracts for the kidney genes (**A**) *Cyp27b1* and (**B**) *Cyp24a1* were derived from a previous genome-wide analyses of mouse kidney cortex in vivo as in Reference [29].

As discussed in the Introduction, 1,25(OH)_2_D_3_ also plays a striking role together with PTH and FGF23 in modulating the expression of *Cyp27b1* and *Cyp24a1* in the kidney. The *Cyp27b1* gene is induced by PTH but suppressed by 1,25(OH)_2_D_3_ and FGF23, while the *Cyp24a1* gene is reciprocally regulated by each of these three hormones [29,90]. This reciprocal regulation enables the activities of these two genes to acutely and dynamically control the levels of 1,25(OH)_2_D_3_ secretion into the blood. The basal and regulated expression of *Cyp27b1* and *Cyp24a1* is mediated by each of the three hormones via a complex regulatory module located within introns of two upstream genes adjacent to the *Cyp27b1* locus and in an intergenic location downstream of *Cyp24a1*, as seen in Figure 4A,B [29,30,90]. These regulatory sites at both gene loci bind both CREB and VDR and mediate the actions of PTH and 1,25(OH)_2_D_3_, respectively. These same enhancers also mediate the actions of FGF23, although the factor responsible for this hormone’s activity is still unidentified. Most importantly, the entire intronic regulatory module at *Cyp27b1* and some segments of the intergenic regulatory complex downstream of *Cyp24a1* are unique to the kidney due to the presence of novel open chromatin structures. These open chromatin modules are absent in non-renal tissues, with the exception that several components of the *Cyp24a1* module that mediates 1,25(OH)_2_D_3_ action are retained across all tissues [30]. Accordingly, removal of the intronic *Cyp27b1* regulatory module eliminates basal and regulated *Cyp27b1* expression selectively in the kidney, while removal of the downstream *Cyp24a1* regulatory module eliminates basal, PTH, and FGF23-regulated expression selectively in the kidney. 1,25(OH)_2_D_3_ regulation of *Cyp24a1* is retained in all tissues. Interestingly, treatment by PTH also leads to a striking enhancement of H3K27 acetylation (H3K27ac) at CREB-binding sites in the *Cyp27b1* gene locus in the kidney but suppresses H3K27ac at CREB-binding sites in the *Cyp24a1* locus (Figure 4A,B). Thus, H3K27ac levels track uniquely with the upregulation of *Cyp27b1* RNA transcripts and the downregulation of *Cyp24a1* RNA transcripts in the kidney. Treatment with 1,25(OH)_2_D_3_, in contrast, leads to a striking suppression of H3K27ac at VDR binding sites in the *Cyp27b1* gene locus in the kidney, but strongly induces H3K27ac at VDR binding sites in the *Cyp24a1* locus. Thus, again, H3K27ac levels correlate uniquely with *Cyp27b1* and *Cyp24a1* RNA transcript regulation in the kidney. As a suppressor of *Cyp27b1* and an inducer of *Cyp24a1*, FGF23 actions on H3K27ac mimic that of 1,25(OH)_2_D_3_ at the genes for both *Cyp27b1* and *Cyp24a1*. Therefore, similarly to 1,25(OH)_2_D_3_, FGF23-regulated H3K27ac levels track uniquely with the downregulation of *Cyp27b1* and the upregulation of *Cyp24a1* transcripts as well. Consistent with the absence of these modules in *Cyp27b1*, no changes in H3K27ac are seen in non-renal tissues. However, 1,25(OH)_2_D_3_ does induce H3K27ac at selected sites in the *Cyp24a1* gene in both renal and non-renal tissues. Finally, detection of H3K36 tri-methylation (H3K36me3) levels increase in response to PTH across the *Cyp27b1* gene body but are suppressed in response to 1,25(OH)_2_D_3_ and FGF23, while detection of this same histone modification decreases in response to PTH but is increased in response to 1,25(OH)_2_D_3_ and FGF23 across the *Cyp24a1* gene body. Additional factors linked to the induction or suppression of these two genes are also detected at each of the sites of regulation in both genes.

## 8. Vitamin D Analogues

Exploring the effects of synthetic analogues of vitamin D using this combination of in vitro and in vivo approaches has the clear potential of revealing molecular differences relevant to unique analogue action. These differences could emerge with authentic interacting factors such as coregulators specific for individual gene targets under investigation if an analogue exerts its actions via a structural alteration that provokes a functional change in the VDR. It is now clear that many of the original hypotheses regarding the basis for coregulator recruitment at genes derived from traditional transfection methods have not been entirely correct. Since current studies are routinely conducted on a genome-wide basis, genes selected for their specific function in Ca and P homeostasis in bone could provide a focus for designing (SAR), identifying and understanding analogues that exhibit non-calcemic actions in an endogenous context relative to 1,25(OH)_2_D_3_. It seems likely that a vitamin D antagonist would reveal itself rapidly under this type of molecular scrutiny as well.

## 9. Conclusions

The purpose of this manuscript has been to delineate new approaches to the study of mechanisms related to the transcriptional regulation of genes by 1,25(OH)_2_D_3_ and their potential application to the analysis of the vitamin D mediated mechanisms in vitro and in vivo. Based on new techniques that involve ChIP-seq analysis, sites of action of the vitamin D hormone can be identified via the co-localization of the VDR and its partner RXR on endogenous DNA on a genome-wide basis in both culture cells and in tissues in vivo. Detection of gene-specific coregulators as well as the activities of these factors via epigenetic changes to histones may also be assessed. The identity of facilitator proteins that initiate the recruitment and/or distribution of RNA polymerase II across the gene body that result in nascent transcripts could also be examined. The ability to detect each of these component proteins and their activities at early time points in a largely gene-specific manner using the vitamin D hormone, as a one-time control suggests that similar studies could be used to characterize the actions of novel vitamin D analogues. This could readily be accomplished to conduct SAR based on newly synthesized compounds as well as to assess the unique mechanistic bases for unusual vitamin D analogue specificities. Indeed, this process could facilitate either the detection and/or mechanisms through which an analogue might retain a non-calcemic activity directly in vivo.

## Figures and Tables

**Table 1:** Overarching Principles of Vitamin D Action in Target Cells (Ref. [45]).

**Active Transcription Unit for Induction: The VDR/RXR heterodimer**
**VDR Binding Sites (The VDR Cistrome): 2000–8000 1,25(OH)_2_D_3_-sensitive binding sites/genome whose number and location are chromatin dependent and a function of cell-type**
**Mode of DNA Binding: Predominantly, but not exclusively, 1,25(OH)_2_D_3_-dependent**
**VDR/RXR Binding Site Sequence (VDRE): Induction mediated by classic hexameric half-sites (AGGTCA) separated by 3 base pairs; Repression mediated by divergent sites**
**Distal Binding Site Locations: Dispersed in *cis*-regulatory modules (CRMs or enhancers) across the genome; located in a cell-type specific manner near promoters, but predominantly within introns and distal intergenic regions; frequently located in clusters of elements**
**Epigenetic CRM Signatures: Defined by the dynamically regulated post-translational histone H3 and H4 modifications**
**Modular Features of CRMs: Contain binding sites for multiple transcription factors that facilitate either independent or synergistic interaction**
**VDR Cistromes: Dynamic alterations in the cellular epigenome during differentiation, maturation, and disease provoke changes to the VDR cistrome that qualitatively and quantitatively affect the vitamin D regulated transcriptome**
